# Supplementary material for: Cyclopia intermedia (Honeybush) Induces Uncoupling Protein 1 and Peroxisome Proliferator-Activated Receptor Alpha Expression in Obese Diabetic Female db/db Mice
Source: Int J Mol Sci. 2023 Feb 15;24(4):3868. doi: 10.3390/ijms24043868 (PMC9964215; doi:10.3390/ijms24043868)
Supplement: Supplementary file 1 [file ijms-24-03868-s001.zip › ijms-2132227-supplementary.pdf]

Supplementary Material

Cyclopia intermedia (honeybush) induces uncoupling protein 1 and peroxisome proliferator-activated receptor alpha expression in obese diabetic female db/db mice

Babalwa Unice Jack [1,*], Pritika Ramharack [1,2], Christiaan Malherbe [3,†], Kwazi Gabuza [1], Elizabeth Joubert [3,4] and Carmen Pheiffer [1,5,6]

[1] Biomedical Research and Innovation Platform, South African Medical Research Council, Tygerberg, Cape Town 7505, South Africa
[2] Pharmaceutical Sciences, School of Health Sciences, University of KwaZulu-Natal, Westville Campus, Durban 4001, South Africa
[3] Plant Bioactives Group, Post-Harvest and Agro-Processing Technologies, Agricultural Research Council (ARC), Infruitec-Nietvoorbij, Stellenbosch 7599, South Africa
[4] Department of Food Science, University of Stellenbosch, Matieland, Stellenbosch 7602, South Africa
[5] Centre for Cardio-Metabolic Research in Africa (CARMA), Division of Medical Physiology, Faculty of Medicine and Health Sciences, University of Stellenbosch, Tygerberg, Cape Town 7505, South Africa
[6] Department of Obstetrics and Gynaecology, Faculty of Health Sciences, University of Pretoria, Pretoria 0001, South Africa
[*] Correspondence: babalwa.jack@mrc.ac.za; Tel.: +27-219-380336
[†] Current address: Analytical Services, Afriplex Pty Ltd, Paarl 7620, South Africa

Validation of molecular docking of PPARα

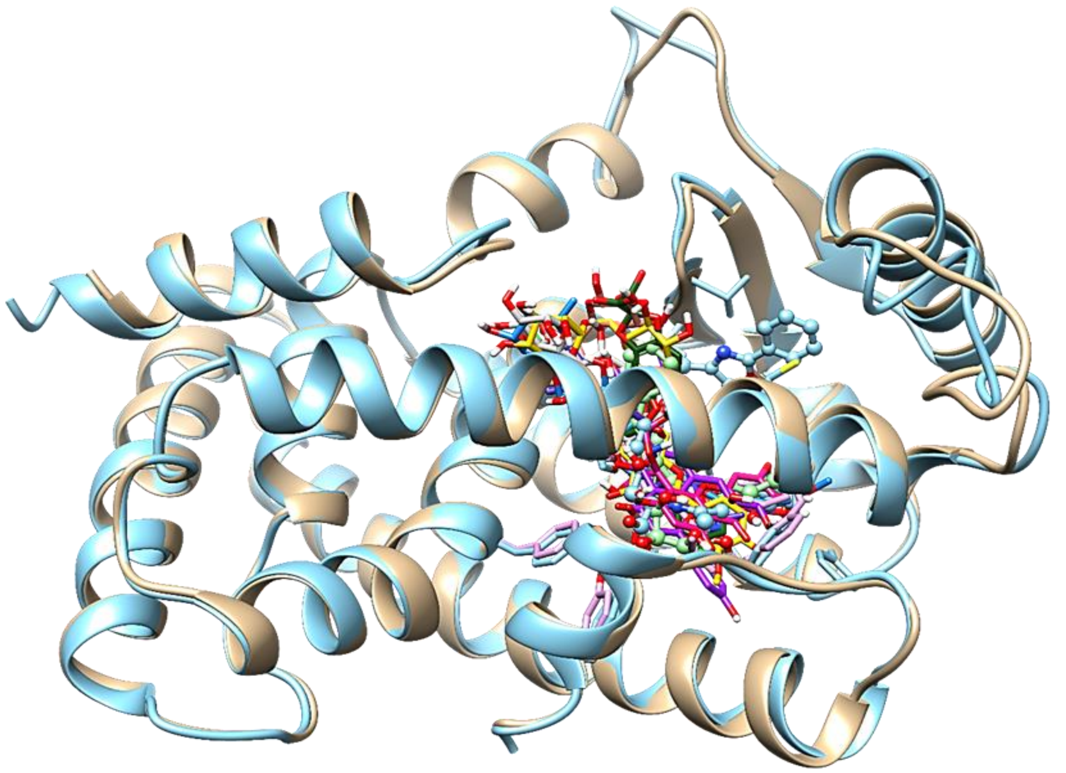

Figure S1: Validation of molecular docking of PPARα by superimposing the complexed models with the crystal template structures. All ligands docked at the same active site to the complexed crystal structures (Blue ribbon: 3DZY- ball and stick ligand, Green: 1K7L- ball and stick ligand, Tan: PPARα model).
